# The Efficacy of Dexmedetomidine alone or with Melatonin on Delirium after Coronary Artery Bypass Graft Surgery: A Randomized Clinical Trial

**DOI:** 10.5812/aapm-138317

**Published:** 2023-08-22

**Authors:** Fatemeh Javaherforooshzadeh, Abbas Babazadeh Dezfoli, Amal Saki Malehi, Behnam Gholizadeh

**Affiliations:** 1 Department of Anesthesia, Pain Research Centre, Ahvaz Jundishapur University of Medical Sciences, Ahvaz, Iran; 2 Pain Research Centre, Ahvaz Jundishapur University of Medical Sciences, Ahvaz, Iran; 3 Department of Biostatistics and Epidemiology, Pain Research Centre, School of Health, Ahvaz Jundishapur University of Medical Sciences, Ahvaz, Iran; 4 Department of Cardiac Surgery, Pain Research Centre, Ahvaz Jundishapur University of Medical Sciences, Ahvaz, Iran

**Keywords:** Postoperative Delirium, Melatonin, Dexmedetomidine, Coronary Artery Bypass Graft, Cardiac Surgery, Elderly Patients

## Abstract

**Background:**

One of the most common cognitive disorders after major surgery is delirium which can increase morbidity and mortality. This study compared the effect of dexmedetomidine with or without melatonin to reduce delirium following coronary artery bypass graft (CABG) surgery.

**Methods:**

This trial was a double-blind, randomized, controlled clinical trial. Eighty patients in two different groups with the administration of dexmedetomidine alone or with melatonin undergoing CABG surgery in Golestan Hospital, Ahvaz, 2022 - 2023, were randomly allocated. This study evaluated the occurrence, onset, and length of delirium, haloperidol, the time required for weaning, and the duration of stays in the intensive care unit (ICU) and hospital.

**Results:**

The occurrence of delirium was lower in the melatonin/dexmedetomidine group (15%) than in the dexmedetomidine group (30 %) (P = 0.09). Additionally, the melatonin/dexmedetomidine group had a significantly lower duration of delirium than the dexmedetomidine group (1.95 (0, 20) and 8.46 (0, 40) P = 0.04). However, no significant difference was observed in the onset of delirium between the two groups (P = 0.25). The length of hospital stays in the melatonin/dexmedetomidine group was significantly shorter than in the dexmedetomidine group (7.53 (7, 10) and 8.60 (7, 15), P = 0.03). However, the two groups demonstrated no significant difference between extubation (P = 0.38) and length of ICU stay (P = 0.19).

**Conclusions:**

The administration of melatonin and dexmedetomidine reduced the incidence of post-cardiac surgery delirium, shortened its duration, and decreased the impact of many risk factors observed in those not receiving the added melatonin.

## 1. Background

Delirium, an acute change in consciousness, attention, cognition, and perception, is one of the most prevalent complications of surgery among elderly patients (1, 2) and can increase morbidity and mortality (3). Although the prevalence of delirium after cardiac surgery is 20 - 60% (4), other factors are involved in its development, including cognitive impairment, advanced age, underlying primary cerebral disease, prolonged bypass procedure, and postoperative sedative administration (5, 6). Recently, most candidates for cardiac surgery are older than 65, and they undergo more complex surgeries, such as redo surgeries and more valve surgeries, than before 2000 (7). Therefore, these patients are more prone to delirium in the postoperative period.

Melatonin is a pineal gland hormone secreted into the circulation by the circadian rhythm. The initiation, maintenance, and efficiency of sleep can be influenced by melatonin. Moreover, it can control circadian rhythm and sleep regulation in critically ill patients (8). Melatonin administration can decrease the occurrence of delirium after cardiac surgery through pain alleviation and sleep cycle modification without any specific side effects (9). Numerous studies have shown that exogenous melatonin can reduce the incidence and duration of delirium (10).

Dexmedetomidine is one of the most highly selective alpha-2 adrenergic receptor agonists. This medication was initially used for mechanically ventilated intensive care unit (ICU) patients’ sedation (11). In addition, dexmedetomidine has long been used as a postoperative sedative to prevent delirium after cardiac surgery (12, 13). Several meta-analyses have demonstrated that dexmedetomidine substantially reduced the occurrence of delirium, agitation, and confusion (14-16). Generally, dexmedetomidine can be beneficial in decreasing the occurrence of postoperative delirium (POD) in elderly patients undergoing cardiac surgeries.

## 2. Objectives

The current study was carried out to evaluate the effectiveness of combining dexmedetomidine and melatonin to prevent POD following coronary artery bypass graft (CABG) surgery.

## 3. Methods

### 3.1. Ethics Approval and Consent to Participate

This randomized controlled clinical trial was approved by the Anesthesiology and Pain Research Center, Ahvaz Jundishapur University of Medical Sciences, Ahvaz, Iran (IR.AJUMS.REC.1401.047) (IRCTID: IRCT20180909040979N7). Setting and Patients: This study was performed on 80 patients who underwent on-pump CABG surgery in Golestan and Imam Khomeini hospitals, Ahvaz, Iran, from January 2022 to February 2023. Informed consent was obtained from all participants before the operation.

The Inclusion criteria included age ≥ 35 and a candidate for elective CABG. The exclusion criteria included emergency procedures, mental illnesses, ejection fraction (EF) < 30%, chronic renal and liver dysfunctions, prolonged mechanical ventilation, and allergy to melatonin or dexmedetomidine.

**Figure 1.**
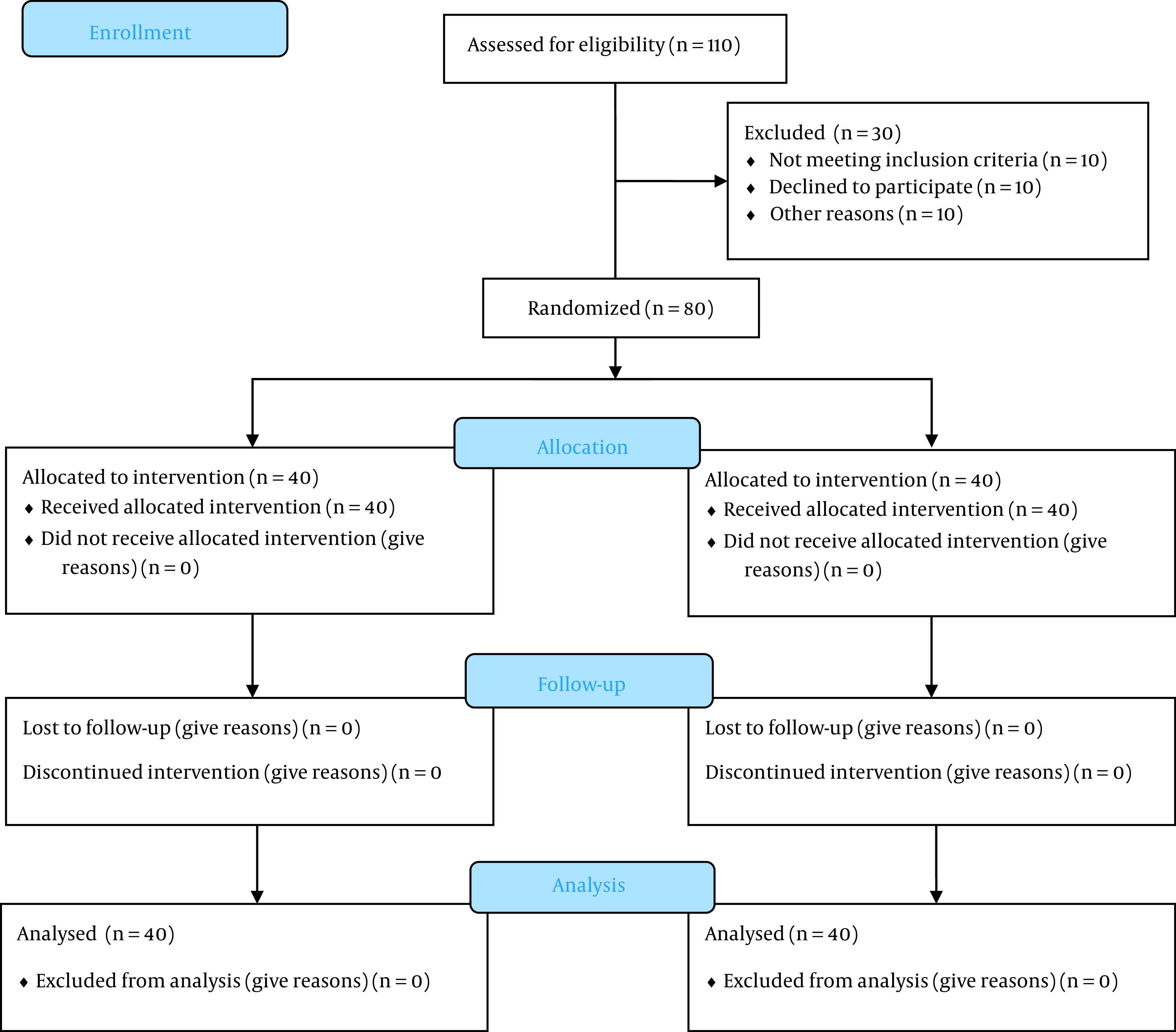
CONSORT diagram shows the process of patient recruitment and allocation. In the present study, 110 cases were included, and 80 were eligible and participated. Patients were randomly assigned to the two groups (each group with 40 cases).

During the study period, 110 subjects who underwent elective on-pump CABG were selected as candidates. After an early assessment, 90 decided to contribute, among whom 10 patients did not meet the inclusion criteria (three patients with EF < 30%, two with off-pump CABG, two with allergy, and three with mental illness). Ultimately, 80 patients were enrolled in the study and were assigned to the two groups with allocation ratio1/1, namely melatonin/dexmedetomidine (A) (n = 40) and dexmedetomidine (B) (n = 40) (Figure 1). 

### 3.2. Randomization and Blindness

If the inclusion criteria were met and none of the exclusion criteria were observed, then the permuted-block randomization technique with a block size of four was employed to randomly assign patients to the melatonin/dexmedetomidine (A) and dexmedetomidine (B) intervention groups. To achieve the study objective, the patients, surgeons, and investigators were blinded to the identity of the treatment group. Moreover, the individuals above were unaware of the type of injectable medication and the surgeries performed by the same surgeon (the researcher conducted the preparation of the injectable medication named A and B).

### 3.3. Sample Size

After a review of the published literature, the incidence of delirium was assumed to be 32% in the control group (17, 18). The recruitment of 74 patients was needed to detect a clinically relevant 25% reduction in the delirium event rate (0.05 and 80% power) with a 1:1 randomization for the entire sample. Considering a 10% dropout rate, the sample size required was 80 (40 per group).

### 3.4. Anesthesia and Cardiopulmonary Bypass Approach

The anesthetic approach was consistent for all patients to minimize its impact on the results. Premedication was achieved according to the hospital design, which included 0.1 mg/kg intramuscular morphine sulfate. All routine monitoring tests (e.g., electrocardiography, pulse oximetry, and noninvasive and invasive blood pressure monitoring) were performed in the operating room.

Anesthesia was induced using midazolam 0.025 mg/kg, fentanyl 10 µg/kg, etomidate 0.15 mg/kg, and pancuronium 0.1 mg/kg. After induction of anesthesia and intubation, central venous cannulation was performed through the internal jugular vein. Anesthesia maintenance was achieved by the infusion of midazolam (0.15 mg/kg/hour), fentanyl (5 µg/kg/hour), and atracurium (0.25 mg/kg/hour) during surgery. Heparin administration, cardiopulmonary bypass (CPB) approach, and surgery were all consistent for every patient. After weaning from CPB, protamine sulfate was administered for heparin reversal. The patients were transported to the cardiovascular intensive care unit (CVICU) after the operation.

### 3.5. Intervention

The patients in the CVICU were randomly assigned to the melatonin/dexmedetomidine group (A) and dexmedetomidine group (B). The subjects in group A received a 3-mg melatonin tablet at 10 p.m. the night before the operation, and the same dose was repeated every night at 10 p.m. for 5 days after the operation. In the intubated patient, melatonin was prescribed through gavage.

In the CVICU, both groups were given Dexmedetomidine 0.5 µg/kg as a bolus; the patients in two groups received a bolus of over 30 minutes, followed by the infusion of 0.3 - 0.5 µg/kg/hour for a maximum of 24 hours which was tapered within 24 hours. If the patient was hemodynamically unstable, the bolus dose was stopped, the infusion of the sedative propofol (0.5 mg/kg/h) was commenced, and the patient was excluded. The Riker Sedation-Agitation Scale (SAS) was used to evaluate the sedation level (19). Dexmedetomidine infusion was adjusted according to SAS score ≥ 3.

### 3.6. Outcomes

The primary outcomes included the occurrence of delirium (assessed preoperatively and at 12-hour intervals for 7 days postoperatively) by the confusion assessment method at the ICU (CAM-ICU) in the intubated patients (20, 21) and after extubation by the CAM. If the patient with delirium was restless, the rate of dexmedetomidine administration was increased, and if the restlessness was severe, haloperidol was used as a 2 - 5 mg intravenous injection. The secondary outcomes were the onset and duration of delirium, haloperidol usage, weaning time, and ICU stay time.

### 3.7. Statistical Analysis

The quantitative data were described as mean ± standard deviation. The qualitative data were expressed as frequencies and percentages. The chi-square test was employed to compare qualitative variables between the two groups. The independent *t*-test and Mann-Whitney U test were utilized to compare quantitative variables between the two groups. Multiple logistic regressions were used to evaluate the impact of melatonin on the occurrence of delirium as the primary outcome by controlling confounder variables. SPSS software (version 22) was utilized for statistical analysis. A P-value < 0.05 was considered statistically significant.

## 4. Results

This study was performed on 110 patients from January 2022 to February 2023. In the current study, 30 ineligible patients were excluded, and 80 continued. Figure 1 illustrates the flowchart of the recruitment and retention of the studied subjects.

The mean age of the patients was 59.76 years (range: 35 - 80). In the present study, 19 (23.7%) and 61 (76.3%) patients were female and male, respectively. No significant differences were observed between the two groups in all baseline characteristics (Table 1). More than half of the participants in the two groups were males and smokers. Furthermore, 87.5% of the patients in the two groups had hypertension. About half of the participants (50% and 62.5% in the Dexmedetomidine and melatonin/dexmedetomidine groups) had diabetes mellitus. Most patients in both groups used angiotensin-converting enzyme inhibitors, statins, and aspirin. No significant difference was noticed between the two groups in past medical and drug history characteristics (Table 2). 

**Table 1.** Continuous Baseline Characteristics in the Dexmedetomidine Group and Melatonin/Dexmedetomidine Group

Variables	Dexmedetomidine Group	Melatonin/Dexmedetomidine Group	P-Value
**Age, y ** ^**a**^	60.08 ± 11.41	59.45 ± 8.45	0.78
**Body mass index kg/m** ^**2**^ ^**b**^	25.46 ± 2.2	26.06 ± 2.23	0.18
**Ejection fraction ** ^**b**^	45.0 ± 6.70	44.5 ± 6.28	0.55
**New York Heart Association score ** ^**b**^	2.23 ± 0.43	2.25 ± 0.44	0.79
**European System for Cardiac Operative Risk Evaluation score ** ^**b**^	1.64 ± 0.29	1.75 ± 0.21	0.11
**Blood urea nitrogen ** ^**b**^	24.73 ± 3.63	24.58 ± 6.06	0.24
**Creatinine ** ^**b**^	1.24 ± 0.22	1.26 ± 0.19	0.49
**Hemoglobin ** ^**b**^	13.63 ± 2.45	13.26 ± 2.49	0.33

^a^ Independent *t*-test

^b^ Mann-Whitney U test

**Table 2.** Categorical Baseline Characteristics in the Dexmedetomidine Group and Melatonin/Dexmedetomidine Group

Variables	Dexmedetomidine Group, No. (%)	Melatonin/Dexmedetomidine Group, No. (%)	P-Value ^a^
**Gender**			0.43
Female	8 (20)	11 (27.5)	
Male	32 (89)	29 (72.5)	
**Smoking**			0.22
Yes	31 (7.5)	26 (65)	
No	9 (22.5)	14 (35)	
**Hypertension **			----
Yes	35 (87.5)	35 (87.5)	
No	5 (12.5)	5 (12.5)	
**Congestive heart failure**			0.69
Yes	3 (7.5)	4 (10)	
No	37 (92.5)	36 (90)	
**Diabetes mellitus**			0.26
Yes	20 (50)	25 (62.5)	
No	20 (50)	15 (37.5)	
**Peripheral vascular disease**			0.46
Yes	13 (32.5)	10 (25)	
No	27 (67.5)	30 (75)	
**Beta-blockers**			0.24
Yes	11 (27.5)	16 (40)	
No	29 (72.5)	24 (60)	
**Calcium channel blockers**			0.26
Yes	25 (62.5)	20 (50)	
No	15 (27.5)	20 (50)	
**Angiotensin-converting enzyme inhibitors**			0.17
Yes	34 (85)	29 (72.5)	
No	6 (15)	11 (27.5)	
**Statins**			0.24
Yes	35 (87.5)	31 (77.5)	
No	5 (12.5)	9 (22.5)	
**Aspirin**			0.24
Yes	35 (87.5)	31 (77.5)	
No	5 (12.5)	9 (22.5)	

^a^ Chi-square test

**Figure 2.**
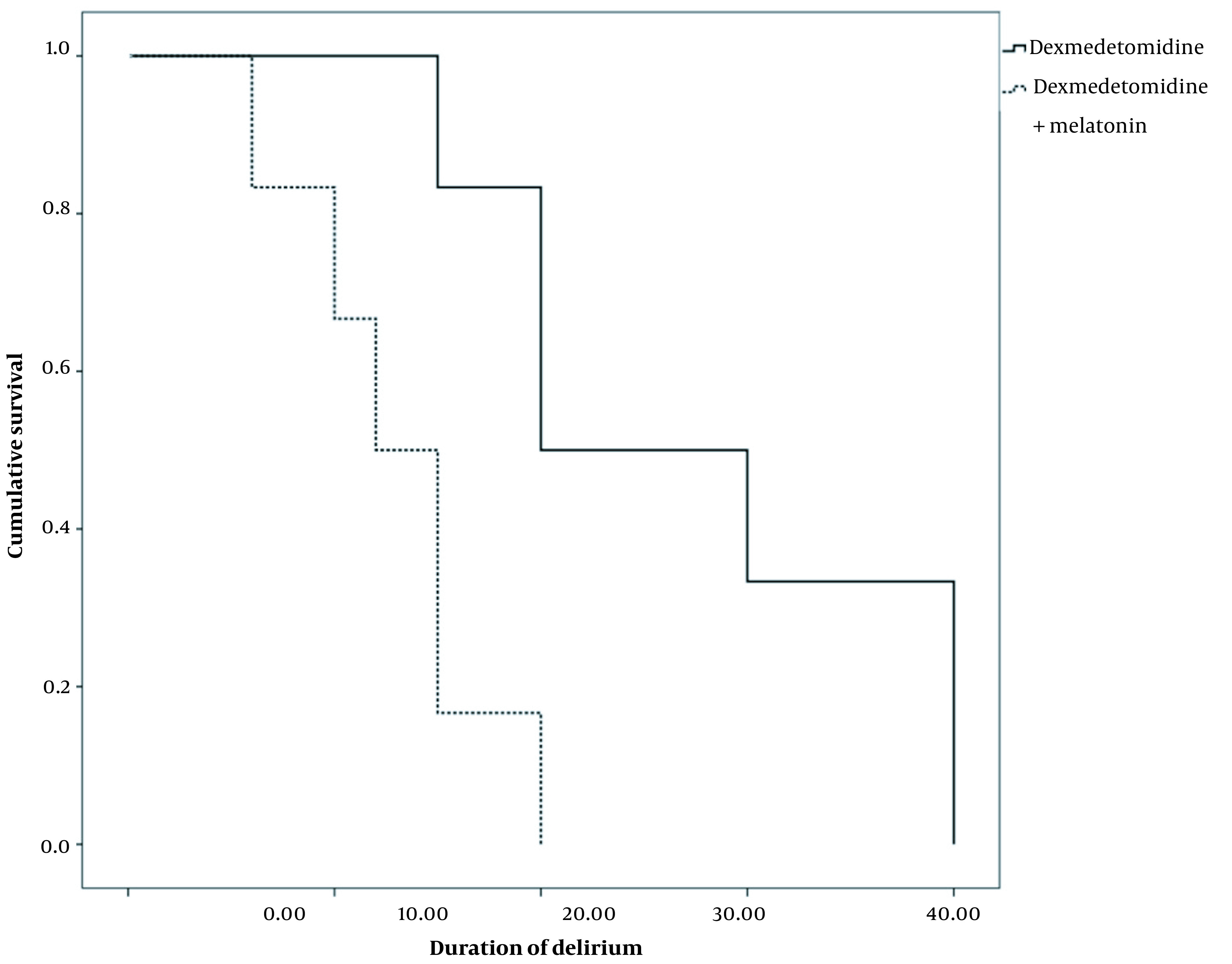
Kaplan-Meier plot for the two groups associated with the duration of delirium

Table 3 compares the primary and secondary outcomes between the two groups. The occurrence of delirium was lower in the melatonin/dexmedetomidine group (6/34) (15%) than in the dexmedetomidine group (12/40) (30%) (P = 0.09). Additionally, the melatonin/dexmedetomidine group had a significantly lower duration of delirium than the dexmedetomidine group (1.95 [0, 20] and 8.46 [0, 40], P = 0.04) (Table 3, Figure 2). Nevertheless, no significant difference was observed in the onset of delirium between the two groups (P = 0.25). The average doses for haloperidol administration were 9.13 and 3.38 mg in the dexmedetomidine and melatonin/dexmedetomidine groups, respectively. The length of hospital stay in the melatonin/dexmedetomidine group was significantly shorter than in the dexmedetomidine group (7.53 [7, 10] and 8.60 [7, 15], P = 0.03). Nonetheless, no significant differences were noticed in extubation time (P = 0.38) and length of ICU stay (P = 0.19) between the two groups (Table 3). 

**Table 3.** Primary Outcomes in the Dexmedetomidine Group and Melatonin/Dexmedetomidine Group ^a^

Variables	Dexmedetomidine Group	Melatonin/Dexmedetomidine Group	P-Value
**Occurrence of delirium, No. (%)** ^**b**^			0.09
Yes	12 (30)	6 (15)	
No	28 (70)	34 (85)	
**The onset of delirium ** ^**c**^	4.51 (0, 20)	5.5 (0, 45)	0.25
**Duration of delirium ** ^**c**^	8.46 (0, 40)	1.95 (0, 20)	0.04
**Haloperidol usage, mg ** ^**c**^	9.13 (0, 40)	3.38 (0, 40)	0.06
**Extubation, h ** ^**c**^	8.80 (4, 24)	8.35 (4, 18)	0.38
**Intensive care unit stay time ** ^**c**^	2.72 (2, 5)	2.33 (2, 4)	0.19
**Hospital stay time ** ^**c**^	8.60 (7, 15)	7.53 (7, 10)	0.03

^a^ Values are expressed as rang unless otherwise indicated.

^b^ Chi-square test

^c^ Mann-Whitney U test

Figure 2 shows that the duration of delirium was significantly lower in the group dexmedetomidine and melatonin.

Table 4 tabulates the odds ratio (OR) and 95% CI comparison for the occurrence of delirium in the melatonin/dexmedetomidine and dexmedetomidine groups. Based on the OR, the melatonin/dexmedetomidine group decreased the occurrence of delirium by less than 50% compared to the dexmedetomidine group (by adjusting the confounder variables). In addition, the number of anastomoses (OR = 6.33, P = 0.001), CPB time (OR = 1.15, P = 0.002), cross-clamp time (OR = 1.11, P = 0.004), inotrope/vasopressor usage (OR = 16.38, P < 0.001), reoperation for bleeding (OR = 24.75, P < 0.001), and hypotension (OR = 3.87, P = 0.02) increased the odds of the occurrence of delirium (Table 4). 

**Table 4.** Binary Logistic Regression Models

Variables	OR	95% CI	P-Value
**Model 1**			
**Treatment group**			
DM	0.38	(0.11 - 1.33)	0.13
D	---	-	
**Number of anastomoses**	6.33	(2.0919.18)	0.001
**Model 2**			
**Treatment group**			
DM	0.29	(0.08 - 1.03)	0.06
D	---	-	
**Cardiopulmonary bypass time**	1.15	(1.05 - 1.26)	0.002
**Model 3**			
**Treatment group**			
DM	0.43	(0.13 - 1.43)	0.17
D	---	-	
**Cross-clamp time **	1.11	(1.03 - 1.18)	0.004
**Model 4**			
**Treatment group**			
DM	0.26	(0.07 - 0.94)	0.04
D	---	-	
**Inotrope/vasopressor usage**			
Yes	16.38	(3.87 - 69.34)	<0.001
No			
**Model 5**			
**Treatment group**			
DM	0.39	(0.13 - 1.19)	0.09
D	---	-	
**Intra-aortic balloon pump**			
Yes	3.73	(0.20 - 67.98)	0.37
No	---	-	
**Model 6**			
**Treatment group**			
DM	0.36	(0.09 - 1.46)	0.15
D	---	-	
**Reoperation for bleeding**			
Yes	24.75	(6.22 - 98.50)	< 0.001
No	---	-	
**Model 7**			
**Treatment group**			
DM	0.42	(0.13 - 1.31)	0.13
D	---	-	
**Hypotension**			
Yes	3.87	(1.21 - 12.46)	0.02
No	---	-	
**Model 8**			
**Treatment group**			
DM	0.45	(0.15 - 1.4)	0.17
D	---	-	
**Bradycardia**			
Yes	2.70	(0.89 - .14)	0.08
No	---	-	
**Model 9**			
**Treatment group**			
DM	0.38	(0.13 - 1.17)	0.09
D	---	-	
**New arrhythmia**			
Yes	1.27	(0.384.24)	0.69
No	---	-	
**Model 10**			
**Treatment group**			
DM	0.41	(0.13 - 1.26)	0.12
D	---	-	
**Hyperglycemia**			
Yes	6.95	(0.56 - 85.92)	0.13
No	---	-	

Abbreviations: DM, melatonin/dexmedetomidine group; D, dexmedetomidine group; OR, odds ratio; CI, confidence interval.

Baseline data that affect the onset of delirium, such as the administration of inotropic auditory disturbance, were compared between the 2 groups. There were no differences between them.

## 5. Discussion

In the present study, it was shown that in the melatonin/dexmedetomidine group, the occurrence of delirium was lower (15% and 30%). In addition, the melatonin/ dexmedetomidine group had a significantly lower duration of delirium than the dexmedetomidine group (1.95 [0, 20] and 8.46 [0, 40], P = 0.04). Nevertheless, the two groups had no significant difference in the onset of delirium (P = 0.25).

The average needed doses for haloperidol administration were 9.13 and 3.38 mg in the dexmedetomidine and melatonin/dexmedetomidine groups, respectively. Regarding the extubation time and ICU stay time, there was no difference between the two groups; however, there was a significant difference between the groups in hospital stay, and it was longer in the dexmedetomidine group than in the melatonin/dexmedetomidine group (P = 0.03).

The difference in the incidence of POD depends on the study’s methodology and mainly on the diagnostic methods of delirium and the characteristics of the study participants. The frequency of delirium after cardiac surgery can reach up to 20 - 60% (22). In this study, delirium was assessed using the CAM-ICU and CAM in the surgical ward after discharge from the ICU. When conducting studies on validity and reliability, the CAM-ICU test has shown a sensitivity of 95 - 100% and a specificity of 89 - 93% (23).

In various studies, dexmedetomidine and melatonin have been used separately to manage POD. A study was conducted on 118 adults undergoing cardiac surgery who were assigned to dexmedetomidine, propofol, or midazolam groups randomly for postoperative sedation after cardiac surgery. The delirium incidence in patients who received dexmedetomidine was significantly lower (3%) than in the other two groups (50%). Participants who developed POD reported significantly longer ICU and hospital stays (24).

In another study by Shi et al., 164 patients were evaluated for receiving the intravenous infusion of propofol with or without dexmedetomidine during cardiac surgery. Propofol was used for postoperative sedation in the ICU. The POD was measured during the first to fifth postoperative days. The difference between the two groups in the incidence of POD was not significant (39.3% and 26.3%, respectively). In the dexmedetomidine group, the average delirium onset time was longer (the second day) than in the propofol group (the first day). Additionally, in the dexmedetomidine group, delirium duration was shorter (2 days) than in the propofol group (3 days) (25). The incidence of delirium in the study above was higher (39.3%) than the results of the current study (31.5%); nonetheless, the delirium onset and duration in patients receiving dexmedetomidine could be compared to the results of the present study. The higher incidence of delirium in the study above in comparison to the current study might be explained by the fact that propofol was utilized for sedation after surgery in both groups.

In another trial performed on 183 patients undergoing cardiac surgery, dexmedetomidine, and propofol were compared upon admission to the ICU. Delirium assessment was carried out at 12-hour intervals within the 5 days after surgery. The POD incidence rates were 17.5% and 31.5% in the dexmedetomidine and propofol groups. The POD onset was on the second and first days after surgery in the dexmedetomidine and propofol groups, respectively. Moreover, the POD duration was 2 and 3 days in the dexmedetomidine and propofol groups, respectively (26). The delirium incidence in the study above (17.5%) was lower than the results of the present study (30.8%), which can be due to propofol usage.

Panagiotis Artemiou’s study investigated the effect of melatonin on the prevention and curative supplementation of POD in patients undergoing CABG. A total of 50 patients were assigned to melatonin and control groups. They discovered that prophylactic melatonin decreased the POD incidence from 28% to 8%. The numbers of treated patients after melatonin therapy were 100% and 42.9% in the melatonin and control groups, respectively (27). The delirium occurrence in the study above (8.4%) was lower than the results of the present study (15%). In the current study, ICU stay was not prolonged due to delirium. The reason for the differences between the results of the study above and the present study might be that the study above was performed on emergency patients, and the researchers administered melatonin without dexmedetomidine.

Another study carried out on 140 patients that underwent CABG to evaluate the effect of melatonin on delirium incidence compared melatonin to a placebo. A significant difference was observed between the two groups in the incidence of the CAM-ICU results on the surgery day and 3 days following the operation; accordingly, the melatonin group had a lower delirium occurrence than the other group (28), which is in line with the findings of the present study.

A meta-analysis of 1714 patients reported lower delirium incidence in older adults using perioperative melatonin. Based on the results, using melatonin and ramelteon was associated with a significantly lower POD incidence in adult subjects undergoing cardiac surgery (OR = 0.46; 95% CI: 0.29 - 0.74; P = 0.001). The subgroup analyses demonstrated that 3-mg melatonin (OR = 0.37; 95% CI: 0.18 - 0.76; P = 0.007) and 5-mg melatonin (OR = 0.34; 95% CI: 0.21 - 0.56; P < 0.001) significantly decreased POD incidence (29). The findings above are in line with the findings of the current study.

Mahrose et al. compared dexmedetomidine with and without melatonin administration in the incidence of postoperative delirium. They observed that the delirium incidence was significantly lower, the onset was significantly more delayed, and the duration was significantly shorter in the dexmedetomidine/melatonin group than in the dexmedetomidine group (30). The results of the aforementioned study align with the present study’s results.

### 5.1. Limitations

In some cases, acute-onset inattentiveness, disorganized thinking, or altered level of consciousness might have been missed during daily delirium assessments, potentially resulting in misclassifying of outcomes. However, generally, routine testing once a day is an accepted method of delirium assessment.

### 5.2. Conclusions

The administration of melatonin with Dexmedetomidine can reduce the incidence of post-cardiac surgery delirium, shortens its duration, and mitigates the impact of many risk factors observed in those not undergoing melatonin treatment.

## Data Availability

The dataset presented in the study is available on request from the corresponding author during submission or after publication.

## Clinical Trial Registration Code:

IRCT20180909040979N7 (2022-05-24)
